# Mentor communication skills training: development, feasibility, and preliminary efficacy

**DOI:** 10.1186/s12909-024-05616-7

**Published:** 2024-06-10

**Authors:** Smita C. Banerjee, Patricia A. Parker, Jessica M. Staley, Ruth Manna, Cassidy Mahoney, Laura Liberman

**Affiliations:** Associate Attending Behavioral Scientist, Department of Psychiatry and Behavioral Sciences, Memorial Sloan Kettering Cancer Center, 633 3rd Avenue, 4th Floor, New York, 10017 USA

**Keywords:** Academic medicine, Cancer care, Communication skills training, Faculty, Mentoring, Mentors, Training evaluation

## Abstract

**Background:**

Mentoring is vital to career development in academic medicine, and communication underlies all aspects of the mentoring relationship. Although training research mentors has been shown to be effective, few academic medicine faculties have received training in how to mentor. The investigators developed a novel intervention, the Mentor Communication Skills Training for Oncology Faculty (“Comskil Mentor Training”) and examined feasibility and preliminary efficacy.

**Methods:**

The study was a single arm pre-post intervention design. The intervention (Comskil Mentor Training) was offered in one virtual 3-hour session and included a didactic lecture with exemplary skill demonstration videos, facilitator-led small group role plays with trained actors, and evaluation. 19 faculty members from 12 departments participated in the training.

**Results:**

All participants completed the training. Overall, the training was rated favorably, with more than 80% of participants indicating that they “agreed” or “strongly agreed” with training evaluation. From pre- to post-training, significant improvement was seen in participants’ overall self-efficacy to communicate with mentees, as well as participants’ overall use of communication skills and mentoring-specific language.

**Conclusions:**

Our findings support the feasibility and preliminary efficacy of a virtually delivered experiential mentor communication skills training program for multidisciplinary clinical and research faculty in oncology.

## Mentor communication skills training: development, feasibility, and preliminary efficacy

Mentoring is vital to career development in academic medicine, and communication underlies all aspects of the mentoring relationship [1, 2]. The National Academy of Sciences, Engineering, and Medicine (NASEM) defines mentorship as “a professional, working alliance in which individuals work together over time to support the personal and professional growth, development, and success of the relational partners through the provision of career and psychosocial support [3].” Mentoring is a learned skill, with training curricula, standardized competencies, and validated assessments [3]. Although mentor training has been shown to be effective, few faculty have been trained in how to mentor [3, 4]. 

In *Entering Mentoring*, the most studied and validated training for mentors of science, technology, engineering, mathematics, and medicine (STEMM) researchers, effective communication is considered a core mentoring competency, along with aligning expectations, assessing understanding, addressing equity and inclusion, promoting professional development, and fostering independence [5, 6]. *Entering Mentoring* was developed to train mentors of researchers, not clinicians [7]. Mentoring may combat burnout, a relevant issue for clinicians and for faculty in the discipline of oncology [8, 9]. No prior mentor trainings, to our knowledge, have been validated in these individuals.

To address this gap, the investigators have developed a novel experiential intervention, the Mentor Communication Skills Training for Oncology Faculty (“Comskil Mentor Training”). Based on other successfully implemented communication skills trainings by the team [10–13], we hypothesize that training oncology clinical and research mentors in relevant communication skills may enhance their self-efficacy and performance as mentors. This paper describes the development of the Comskil Mentor Training and evaluates its feasibility and preliminary efficacy.

## Methods

### Participants

Participants were academic medicine faculty at a comprehensive cancer center. Selection of participants was based on convenience sampling, with attention to interest in the training; representation of different departments, career trajectories, and career stages; and having some mentoring experience. The participant’s career path was as either focused on clinical care or research based on how most (> 50%) of their time was allocated. Additional faculty data (demographics, number of mentees, work location) were provided by self-report. The training and evaluation reported in this paper received exemption from Memorial Sloan Kettering Institutional Review Board (Protocol Number X21-049), as per 45 CFR 46.104(d)(1). The study did not require informed consent from participants. Oncology faculty members participated voluntarily in the study following the invitation sent by the last author (LL). The participants indicated interest and availability via email, and this agreement to participate was documented.

### Comskil mentor training curriculum

The Comskil Mentor Training curriculum was developed by engaging in the following sequential steps: (a) literature review, including a review of *Entering Mentoring*, (b) consensus review meetings (with faculty leaders/mentors, researchers, and communication skill experts), (c) modular blueprint development, (d) training methods development, (e) scenario development (for role plays and exemplary video clips), (f) making necessary revisions and adaptations, and (g) assessment of the training module. The training curriculum followed the guidelines of the Comskil Model, a skills-based approach to teaching communication skills in a cancer setting [14]. As per that model, we recommended eight communication *strategies* (a priori plans that direct communication behavior toward the desired communication goal) for mentors to use in guiding mentees: (a) introduction; (b) initial establishment of relationship – develop ground rules of communication, create a safe space, establish rapport; (c) aligning expectations (e.g., logistics, communication, meetings, responsibilities, etc.); (d) assessing mentor and mentee’s skills and needs; (e) setting goals that are SMART: specific, measurable, attainable, relevant, and time-based [15]; (f) providing and eliciting feedback [16]; (g) expressing empathy; and (h) closing the conversation. The *strategies* are accomplished by using communication *skills* (standalone verbal utterances) and *process tasks* (set of verbal and nonverbal behaviors that create a conducive environment for effective communication; Table 1). Although presented consecutively, the strategies can be used, according to the mentor-mentee interaction context. For instance, if a mentee is working on writing their first manuscript for publication, the mentor could focus on Strategy (e) and focus on SMART goal setting with the mentee.

**Table 1:** Comskil mentoring training blueprint

Mentorship Strategies	Skills	Process Tasks
1. Introduction	- Ask open questions - Declare agenda - Invite agenda - Negotiate agenda	- Review mentee CV (should have done prior to meeting) - Make introductions (incl. pronouns and preferred name) - Active listening – focus on mentee - Minimize distractions
2. Initial establishment of relationship (ground rules of communication, create a safe space, establish rapport)	- Check preference for information (and learning styles) - Normalize - Ask open questions - Providing a rationale	- Maintain eye contact - Encourage open communication and participation - Confidentiality -Discuss how the mentoring relationship came to be and prior mentoring experiences -Encourage note-taking -Allow a safe space to discuss differences (racial, gender, perspectives, etc.)
3. Aligning expectations (e.g., logistics, communication, frequency of meetings, etc.)	- Check understanding - Check preference for information - Invite questions - Endorse question asking	- Elicit goals: initial and ongoing - Make partnership statements - Clear misunderstandings, if any - Encourage ongoing bi-directional feedback
4. Assessing mentor and mentee’s skills, needs, and interests	- Take stock - Check understanding - Transition - Summarize - Normalize - Acknowledge - Validate - Ask open questions	- Share your perspective (strengths/weaknesses, interests – and seek theirs) - Explore network (provide connections) -Engage with other mentors, as appropriate
5. Identifying goals/smart goals – **S**pecific, **M**easurable, **A**ttainable, **R**elevant, **T**ime based (SMART)	- Ask open questions - Check understanding - Invite questions - Endorse question asking	- Elicit mentee goals (short term/long-term, structuring ideas…) - Discuss the concept of SMART goal (introduce a compact) - Encourage transparency about accountability - Discussion of potential obstacles in attaining goals
6. Providing and eliciting feedback	- Ask open questions - Check understanding - Acknowledge - Encourage expression of feelings - Clarify - Restate - Provide a rationale - Review next steps	- Encourage transparency - Make specific and timely observations instead of general feedback - Acknowledge differences in perspectives (Third-story modeling) - Communicate a safe space - Allow time to integrate
7. Communicating empathically	- Encourage expression of feelings - Acknowledge - Validate - Normalize - Praise mentee efforts	- Silence - Active listening
8. Closing the conversation	- Summarize content and experience of meeting - Check understanding - Ask open questions - Review next steps	If appropriate: - Offer to talk with other mentors/colleagues - Plan next interaction - Adapt to evolving roles

***Goal*** To provide mentors with communication skills necessary to guide mentees to achieve their objectives, which may include formulating career goals, planning how to achieve career and personal goals, learning skills necessary to succeed in career goals, finding resources, and having a sponsor/champion for their career advancement

### Training format

The Comskil Mentor Training was a 3-hour training in either December 2021 or January 2022. The program was developed and implemented over ZOOM due to the pandemic. Training included a didactic component (45 min), facilitator-led small group role plays (90 min), evaluation (30 min), and a 15-minute break. The didactic component included a lecture and interactive discussion addressing mentoring terminology, importance of mentoring in an academic setting, SMART goals, feedback, barriers to mentoring, and a conversational blueprint to provide mentors with communication skills necessary to guide mentees (Table 1). The blueprint was discussed and provided in digital format to participants. Exemplary videos were embedded into the didactic presentation to illustrate key communication skills.

After the didactic (using the breakout room feature in Zoom), participants were split into groups of three for the role play sessions, which were co-facilitated by Comskil faculty and other MSK faculty mentors. Prior work in communication skills highlights that small-group role sessions, made up of 2–3 learners are usually preferable for skills acquisition as they allow each learner dedicated time in their particular role [17]. Most of the facilitators were faculty who were involved in the development of the Comskil Mentor Training program. All facilitators had previously participated in a Comskil Facilitator Training that teaches our approach to training and feedback, as well as the structure and format of the training sessions. These experiential exercises allowed each participant to practice specific mentoring strategies with a standardized mentee (SM), portrayed by a trained actor, and then to engage in feedback discussion with fellow peer participants and facilitators.

Finally, for evaluation, participants completed post-training surveys and one Standardized Mentee Assessment (SMA). The SMA included a 10-minute video recorded interaction between participant “mentor” and SM on a provided scenario, using standardized scripts for the SM. At the end of the SMA, the participants were thanked for their participation and logged out of the Zoom training.

### Measures

Study measures included training evaluation (post-training), self-efficacy in communication with mentees (pre- and post-training), communication skills uptake and mentoring-specific language use (assessed using SMAs, at both pre- and post-training). The pre-training questionnaires and SMAs were completed within 2 weeks prior to the training, and post-training questionnaires and SMAs were completed immediately after-training.

**Training evaluation** This measure was modeled after prior program evaluation measures created by the study team [13]. Participants completed a post-training evaluation consisting of 15 statements using a 5-point Likert scale with anchors of (1) “strongly disagree” to (5) “strongly agree.” The statements measured post-training attitudes regarding engagement (e.g., “I got easily distracted during the role play”), novelty (e.g., “The role play was different than other mentoring trainings I have participated in”), and reflectiveness (e.g., “This role play made me think about specific things I can do about my communication skills when engaging with my mentees”). A higher score indicated a more favorable evaluation.

**Self-efficacy in communication with mentees** Self-efficacy was modeled after prior self-efficacy measures created by the study team [18]. The measure consists of five items using a 5-point Likert scale with (1) “strongly disagree” to (5) “strongly agree,” such as “I am confident in my ability to guide mentees to formulate career goals”, and “I am confident in my ability to guide mentees in planning how to achieve career and personal goals.” A higher score indicated high confidence.

**Communication skills uptake via standardized mentee assessment (SMA)** Before and after training, each participant completed a SMA, a 10-minute video recorded interaction between participant “mentor” and SM on a provided scenario. The SMA scenario was based on concepts taught in the training, including introduction and aligning expectations. We adapted the established Comskil coding system [19] for mentoring [mentoring Comskil Coding System or mCCS] to code all video recorded SMAs. The mCCS codes presence/absence of verbal utterances (skills) that are present in the mentor-SM interaction but does not code for nonverbal behaviors. The mCCS includes 20 individual skills, grouped under five communication skills categories: agenda setting, checking, questioning, information organization, and empathic communication.

Communication skills uptake was assessed by indicating the presence/absence of each of the 20 skills used in the SMA. We measured the count of how many unique skills were used, regardless of how many times each was used, and could range from 0 to 20. We also created an overall combined score for communication skills by summing together all scores received in the five categories, for pre- and post-training. A higher score indicated more utilization of communication skills in the interaction.

**Mentoring-specific language use via SMA** Based on our blueprint that contains strategies, skills, and process tasks, we developed a checklist of 28 mentoring-specific language uses including: “ask about the mentee’s prior experience (clinical, research, or teaching),” “ask about SMART goals (must mention the term “SMART goal”)”, and “discuss institutional goals.” Coders coded the presence/absence of each mentoring-specific language use. We measured the count of how many unique skills were used, regardless of how many times each was used, and could range from 0 to 28. Summed scores were created; a higher score indicated more utilization of mentoring language in the interaction.

### Coding

Two trained coders coded all the SMA videos using the mCCS. We assessed inter-coder agreement at the beginning of coding and at the midpoint by double coding (i.e., both coders coded the same set of SMAs to ensure agreement) 10% of data. We asked all coders to provide an example utterance for each code and highlighted where there were disagreements. The coders then met with the team to discuss and provide additional training around disagreements or incorrect codes before allowing coders to proceed independently.

### Data analysis

The data were analyzed using SPSS 24 for Windows (IBM Corporation Armonk, New York). For training evaluation, a rating of “agree” or “strongly agree” on each evaluation item was considered as endorsement of the training and was analyzed descriptively. For assessing improvements in self-efficacy, communication skills and mentoring-specific language use respectively, paired t tests were used to assess significant pre-post-training differences. Two-tailed significance tests were used and *p* < .05 was considered statistically significant [11]. In the results, both significance levels and effect sizes (Cohen’s d) are reported (d = 0.2, small; d = 0.5, medium; and d = 0.8, large effect).

## Results

### Participants

Nineteen faculty members from a comprehensive cancer center participated in the 3-hour Comskil Mentor Training. Faculty demographic and other data are shown in Table 2. These 19 faculty members were from 12 departments. Most participants were women, White, non-Hispanic, had at least six mentees, and had attended previous Comskil trainings focused on doctor-patient communication. Almost half were junior (Assistant level) faculty. Of 19 participants, 17 (89%) engaged in both clinical care and research; for almost 2/3 of all participants, time allocation was predominantly clinical.

**Table 2:** Participant sociodemographic characteristics (*N* = 19)

Variable	Range	*N* (%)
Rank	Assistant Attending Associate Attending Attending	9 (47) 6 (32) 4 (21)
Age	M = 45.40, SD = 9.45, Range = 33–67 years	
Gender*	Woman Man	10 (53) 8 (42)
Race*	White Asian Black or African American American Indian or Alaska Native Native Hawaiian or Other Pacific Islander More than one race	11 (58) 2 (11) 1 (5) 0 (0) 0 (0) 4 (21)
Hispanic or Latino*	No Yes	14 (74) 4 (21)
Years of Experience	Less than 1 year 1–5 6–10 11–15 16+	1 (5) 3 (16) 7 (37) 4 (21) 4 (21)
Department	Medicine Surgery Neurology (Others: 1 each from Anesthesiology, Chemical Biology, Laboratory Medicine, Medical Physics, Molecular Pharmacology, Pathology, Psychiatry and Behavioral Sciences, Radiation Oncology, Radiology)	4 (21) 3 (16) 3 (16) 9 (47)
Career focus	Clinical Research	12 (63) 7 (37)
Work Location	NYC Regional Areas (New Jersey, Long Island, Westchester)	15 (79) 4 (21)
Approximate Number of Mentees	1–5 6–10 11–15 16–20 21–25 26+	9 (47) 3 (16) 2 (11) 1 (5) 1 (5) 3 (16)
Past Comskil Training	No Yes	13 (68) 6 (32)

*Missing values

### Training evaluation

Overall, participants rated the Comskil Mentor Training favorably. Specifically, more than 80% of participants indicated that they “agreed” or “strongly agreed” with twelve of the fifteen evaluation items for the role plays with SMs (Table 3). Descriptive results indicated that participants rated the role play more favorably for engagement and reflectiveness, but less favorably for novelty.

**Table 3:** Participant-rated evaluations for comskil mentor training (*N = 19*)

Items from Module Evaluation	M (SD)	Endorsement *N* (%)
**Engagement**	**4.33 (0.53)**	--
1. The role play was interesting to me.	4.58 (0.51)	19 (100%)
2. I got easily distracted during the role play. (R)	1.95 (0.78)	16 (84%)
3. I enjoyed this role play.	4.26 (0.81)	17 (89%)
4. This role play was boring. (R)	1.58 (0.61)	18 (95%)
**Novelty**	**3.94 (0.82)**	**--**
5. I’ve never done anything like what I did in the role play today.	3.47 (1.31)	10 (53%)
6. The role play was different than other mentoring trainings I have participated in.	4.11 (0.96)	13 (68%)
7. The role play was unique.	4.21 (0.92)	17 (89%)
**Reflectiveness**	**4.31 (0.54)**	**--**
8. This role play made me think about the importance of communication skills in mentoring.	4.33 (0.97)	17 (89%)
9. This role play made me think about reasons for making changes in my communication with mentees.	4.26 (0.93)	18 (95%)
10. This role play made me think about specific things I can do with communication skills when interacting with my mentees.	4.47 (0.61)	18 (95%)
11. This role play helped me figure out how I can incorporate communication skills when engaging with my mentees.	4.37 (0.68)	17 (89%)
12. This role play encouraged me to maintain my communication skills when interacting with my mentees.	4.21 (0.79)	17 (89%)
13. This role play provided new information about communication skills and process tasks that I can use with my mentees.	4.42 (0.51)	19 (100%)
14. This role play made me think about my communication skills with my mentees.	4.53 (0.51)	19 (100%)
15. This role play made me think about my peers’ communication skills with their mentees.	4.00 (0.78)	14 (74%)

*Note* Items 1–15 were scored on a 5-point Likert scale with anchors at (1) “Strongly Disagree” to (5) “Strongly Agree.” Endorsement = percentage of participants that endorsed “Agree” or “Strongly Agree” (Items 1–15); exception: Items 2 & 4. For items 2 & 4, Endorsement = percentage of participants that endorsed “Disagree” or “Strongly Disagree”

### Self-efficacy

Participants’ overall self-efficacy to communicate with mentees significantly improved [t(18)=-3.97, *p* < .001] from pre- (M = 3.51, SD = 0.74) to post-training (M = 4.03, SD = 0.37). Each of the five individual self-efficacy items significantly improved from pre- to post-training (Table 4).

**Table 4:** Changes in self-efficacy and communication & mentoring skills from pre- to post-training (*N* = 19)

Self-Efficacy Items	Pre-Training M (SD)	Post-Training M (SD)	t(18)	Cohen’s d	
1. I am confident in my ability to guide mentees to formulate career goals.	3.53 (1.12)	4.16 (0.77)	-2.47*	0.66	
2. I am confident in my ability to guide mentees in planning how to achieve career and personal goals.	3.37 (1.01)	4.11 (0.46)	-3.44**	0.94	
3. I am confident in my ability to guide mentees to learn skills necessary to succeed in career goals.	3.53 (0.91)	4.00 (0.47)	-2.14*	0.65	
4. I am confident in my ability to guide mentees to finding appropriate resources.	3.72 (0.83)	4.00 (0.77)	-2.56*	0.35	
5. I am confident in my ability to guide mentees to find a sponsor/champion for their career advancement.	3.26 (0.87)	3.95 (0.41)	-3.98***	1.01	
**Overall Self-Efficacy**	**3.51 (0.74)**	**4.03 (0.37)**	-3.97***	**0.89**	
**Communication and Mentoring Skills**					
Agenda setting	0.42 (0.51)	0.90 (0.74)	-2.14*	0.75	
Checking	0.00 (0.00)	0.32 (0.58)	-2.36*	0.78	
Questioning	1.63 (0.68)	2.26 (0.93)	-2.47*	0.77	
Information organization	0.26 (0.45)	0.42 (0.61)	-1.84	0.30	
Empathic communication	0.95 (0.71)	1.58 (0.90)	-2.88**	0.78	
**All communication skills**	**3.26 (1.45)**	**5.47 (1.77)**	-5.06***	**1.37**	
Mentoring-specific skills	7.26 (2.81)	10.42 (2.77)	-5.04***	1.13	

*p < .05, **p < .01, ***p < .001 Cohen’s guide for interpreting effect sizes: small effect, d = 0.2; medium effect, d = 0.5; and large effect, d = 0.8

### Communication skills and mentoring-specific language use uptake

Overall use of communication skills improved significantly, t(18)=-5.06, *p* < .001 from pre- (M = 3.26, SD = 1.45) to post-training (M = 5.47, SD = 1.77), with a large effect size for all skill categories (i.e., agenda setting, checking, questioning, and empathic communication) except one (information organization skills). Use of mentoring-specific language also improved significantly [t(18)=-5.04, *p* < .001] from pre- (M = 7.26, SD = 2.81) to post-training (M = 10.42, SD = 2.77). Table 4 presents the change results for both communication skills and mentoring-specific language use, from pre-to-post training.

## Discussion

Effective communication is a core mentoring competency in which “the mentor engages in active listening with the mentee, provides timely and constructive feedback, recognizes that communication styles differ, and works with the mentee to accommodate their personal communication styles [20].” The *Entering Mentoring* training for mentors of STEMM research trainees consists of eight 60-minute facilitated discussions, including one on effective communication, in a collaborative, problem-solving format that includes case studies; this training has been validated in studies including a randomized controlled trial [20–22]. Research mentees and mentors reported improvement in mentors’ skills, knowledge, and behaviors after the training, but the training has not been validated in oncology faculty, including clinicians.

The Comskil Mentor Training has similarities and differences when compared with the communication skills module in Entering Mentoring [5]. Both trainings include facilitated discussions; both teach participants to engage in active listening and provide feedback; and both include small breakout groups to encourage collaboration and problem solving. The aspects of our intervention that differ from *Entering Mentoring* include the role play component, oncology setting, and application of training to clinicians and well as researchers. To our knowledge, our study is the first report of a mentor communication skills training intervention for oncology clinical and research faculty.

The role play component of the Comskil Mentor Training is “hands on”. Learners practice specific mentoring strategies with a standardized mentee (SM) who is a trained actor. The role play approach has been used with trained actors playing the part of standardized patients to train doctors to communicate with patients, but this approach has not been used to train mentors to communicate with mentees. The use of SMs played by trained actors lends itself to pre- and post-intervention assessments so that self-efficacy and use of specific skills can be quantified before and after training.

Among our learners, almost two-thirds were clinicians rather than researchers. Although mentor training has been developed and validated for STEMM researchers, clinicians also need mentoring. Prior work has suggested that clinical faculty may have less access to and satisfaction from mentoring than faculty focused in research, and benefit from formalized mentoring and goal-setting [23]. Clinicians, therefore, may glean particular benefit from enhanced mentoring that our intervention aims to provide.

The virtual (Zoom-based) Comskil Mentor Training was feasible: all participants completed the training and evaluations. The Comskil Mentor Training was favorably received as evidenced through high training evaluation ratings. Participants rated the Comskil Mentor Training as engaging and reflective, but not necessarily novel. This finding was likely due to participants’ prior experiences with Comskil trainings for doctor-patient communication that offered similar didactic and experiential learning opportunities [9]. However, it was encouraging to note that, despite familiarity with communication skills training, participants actively took part in the role plays with SMs, and they indicated that the training stimulated reflection.

The training resulted in significant improvements in mentors’ immediate post-training self-efficacy to use the learned skills, as well as greater use of communication skills and mentoring-specific language in simulated interactions with mentees. The results of video-recorded interactions with SMs before and immediately after training demonstrated improvements in all communication skills categories (agenda setting, questions, checking, and empathic communication) as well as mentoring-specific language use. This finding is particularly impressive because most participants were experienced mentors and many had previous communication skills training with respect to doctor-patient interactions, and yet still demonstrated uptake in communication skills as well as mentoring-specific language use.

Our intervention is timely. Requirements for mentor education are likely to increase, as evidenced by the National Institute of General Medical Sciences (NIGMS) requirement that training grant applications describe how the program faculty are trained to ensure the use of evidence-based mentoring practices [24]. Furthermore, if we are to increase the diversity of the oncology workforce, we must enhance the ability of mentors to communicate with mentees of diverse backgrounds in order to provide culturally responsive mentoring [7]. 

### Limitations of the study

There were some limitations worth noting. This study was implemented at one comprehensive cancer center with a dedicated communication skills training and research lab. As such, the results may not be generalizable. Second, the intervention was offered virtually due to the pandemic; in-person delivery of our intervention has not been evaluated. Third, the study had a small number of participants with limited racial/ethnic diversity; future iterations of the training could include the cultural aspect of mentoring training and a greater focus on training a diverse health care workforce. Fourth, we evaluated improvements in communication skills and mentoring-specific language use immediately after training; the duration of these improvements is unknown. Fifth, our training evaluation was performed with standardized mentees (i.e., trained actors); the impact of Comskil Mentor Training on mentee outcomes has not yet been evaluated. Finally, the training was offered as a single training session; future work may evaluate the benefit of additional booster sessions.

## Conclusion

This paper presents the development, feasibility, and initial efficacy of a mentor communication skills training for clinical and research faculty at a major cancer center. Unlike prior validated trainings, our intervention is in the oncology setting and includes clinicians as well as researchers. Our findings show that virtually-delivered mentor communication skills training at a cancer center is feasible, was evaluated favorably, and has the potential to improve participants’ self-efficacy, communication skills and mentoring-specific language use. Further study is needed with larger, more diverse participant groups and inclusion of other institutions to determine if results are generalizable and to identify factors associated with training efficacy. Additional work is needed to assess whether the training led to sustainable changes in mentoring behavior with actual mentees and the impact of the training on mentee outcomes.

## Data Availability

Data cannot be shared openly but are available on request from authors.

## Abbreviations

NASEM: The National Academy of Sciences, Engineering, and Medicine
STEMM: Science, technology, engineering, mathematics, and medicine
SMART: Specific, measurable, attainable, relevant, and time-based
SM: Standardized mentee
SMA: Standardized Mentee Assessment
mCCS: Mentoring Comskil Coding System
